# Copy‐number variation of *MCL1* predicts overall survival of non‐small‐cell lung cancer in a Southern Chinese population

**DOI:** 10.1002/cam4.774

**Published:** 2016-06-05

**Authors:** Jieyun Yin, Yangkai Li, Hao Zhao, Qin Qin, Xiaorong Li, Jiao Huang, Yun Shi, Shufang Gong, Li Liu, Xiangning Fu, Shaofa Nie, Sheng Wei

**Affiliations:** ^1^ Department of Epidemiology and Biostatistics and MOE Key Lab of Environment and HealthSchool of Public HealthTongji Medical CollegeHuazhong University of Science and TechnologyWuhanChina; ^2^ Department of Epidemiology and BiostatisticsSchool of Public HealthMedical College of Soochow University199 Ren‐ai Road Industrial Park DistrictSuzhouChina; ^3^ Department of Thoracic SurgeryTongji Medical CollegeTongji HospitalHuazhong University of Science and TechnologyWuhanChina; ^4^ Department of Social Science and Public HealthSchool of Basic Medical ScienceJiujiang UniversityNo. 17Lufeng RoadJiujiang332000China

**Keywords:** *BCL2L1*, copy‐number variation, *MCL1*, non‐small‐cell lung cancer, prognosis

## Abstract

*BCL2L1* and *MCL1* are key anti‐apoptotic genes, and critical for cancer progression. The prognostic values of *BCL2L1* and *MCL1* copy‐number variations (CNVs) in non‐small‐cell lung cancer (NSCLC) remain largely unknown. Somatic CNVs in *BCL2L1* and *MCL1* genes were tested in tumor tissues from 516 NSCLC patients in southern China; afterward, survival analyses were conducted with overall survival (OS) as outcome. Additionally, the associations between CNVs and mRNA expression levels were explored using data from 986 NSCLC patients in the Cancer Genome Atlas project. It was found that amplifications of *BCL2L1* and *MCL1* were associated with unfavorable OS of NSCLC, with adjusted hazards ratio of 1.62 (95% confident interval [CI] = 1.10–2.40; *P* = 0.015) and 1.39 (95% CI = 1.05–1.84; *P *=* *0.020), respectively. Amplifications of *MCL1,* but not *BCL2L1,* were related with higher mRNA expression levels of corresponding gene, compared with non‐amplifications (*P *= 0.005). Interestingly, after incorporating with *MCL1* CNV status, clinical variables (age, sex, TNM stage, and surgical approach) showed an improved discriminatory ability to classify OS (area under curve increased from 72.2% to 74.1%; *P *=* *0.042, DeLong's test). Overall, *MCL1* CNV might be a prognostic biomarker for NSCLC, and additional investigations are needed to validate our findings.

## Introduction

An estimated 1.8 million new lung cancer cases occurred in 2012, accounting for about 13% of total cancer diagnoses 1. Lung cancer was the most frequently diagnosed cancer and the leading cause of cancer death among males in 2012. Among females, lung cancer was the leading cause of cancer death in more developed countries, and the second leading cause of cancer death in less developed countries 1. In 2013, lung cancer was the fourth most common cause of death for men and women in China overall 2, and lung cancer is the number one cause of death among people with malignant tumors in China 3. Non‐small‐cell lung cancer (NSCLC) is the most common type of lung cancer. Although treatment (such as surgery, radiotherapy, and/or chemotherapy) has improved the rate of survival of patients with NSCLC in recent years, the long‐term survival rate still has room to improve. TNM classification is the basis for prognostic management of NSCLC; however, it does not provide sufficient information about biological tumor progression 4. There is still demand for revealing biomarkers for patients’ survival. Recent genomic studies revealed potential therapeutic targets for lung cancer, including *ROS1* rearrangements, *MET* amplification, *RET* fusions, and activating mutations in *BRAF*,* HER2*, and *KRAS* in frequencies exceeding 1%^5^. Nevertheless, it is possible that some other unknown genetic factors may also modulate survival outcomes of NSCLC patients.

Studies suggest that Bcl2 family not only plays an important role in resisting cell death but also has function in cell‐cycle control 6. At the same time, it is proposed that the anti‐apoptotic Bcl‐2 family members may participate in the inhibition of autophagy, which is believed to be a nonapoptotic form of programmed cell death 7. Altered expression of members in this family leads to aberrant cell proliferation and malignant growth 8, 9. Study also supports the idea that Bcl2 family proteins represent important therapeutic targets 10. *BCL2L1*, also known as *BCL‐X*, is mapped to chromosome 20. Amplifications of 20q have been previously noted in various cancers 11, 12. BCL2L1 proteins regulate outer mitochondrial membrane channel opening, which in turn regulates mitochondrial membrane potential, and thus controls the release of cytochrome and the production of reactive oxygen species, both of which are potent inducers of programmed cell death 13. It is shown that BCL2L1, but not BCL2, could suppress mitophagy mediated by FUNDC1 through its BH3 domain, eventually mediating mitochondrial quality control that is essential for cell survival 14. *BCL2L1* DNA copy number is increased in colorectal cancers compared to adenomas 15. Myeloid cell leukemia sequence 1 (*MCL1*), is located in 1q21.2. *MCL1* functions as an anti‐apoptotic molecule and is capable of blocking apoptosis induced by various apoptotic stimuli, including etoposide, staurosporine, UV irradiation, calcium ionophore A23187, and c‐Myc overexpression 16. High levels of *MCL1* expression were found in many different cancer types 10, 17. *MCL1* copy number gain is a frequent event in several cancers, like mantle cell lymphoma, lung cancer, and breast cancer 18, 19. What is more, overexpression of *MCL1* has also been reported to be correlated with poor survival and resistance to chemotherapeutic agents 10.

Above all, we can hypothesize that copy‐number variations (CNVs) in *BCL2L1* and *MCL1* may be associated with cancer prognosis. To date and to the best of our knowledge, there have been rare studies addressing the roles of CNVs of *BCL2L1* and *MCL1* in NSCLC outcomes. In this study, we detected *BCL2L1*,* MCL1* CNVs in DNA from NSCLC tumors tissues in a southern Chinese population; additionally, we conducted survival analyses to analyze prognostic values of *BCL2L1*,* MCL1* CNVs on overall survival (OS).

## Materials and Methods

### Ethics statement

The study was approved by the institutional review boards of Tongji Medical College. All patients were informed about the aims of specimen collection and gave signed written consent in accordance with the ethical guidelines of Tongji Medical College.

### Study subjects

All the patients were histopathologically confirmed, without preoperative chemotherapy or radiotherapy, and underwent lung cancer surgery in the Affiliated Tongji Hospital of Huazhong University of Science and Technology from October 2006 to June 2012. Only patients with fully characterized somatic CNV status and intact survival information were included in this study, and there was no selection bias (Table S1). Eventually, 516 patients were recruited, among which 390 patients provided formalin‐fixed paraffin‐embedded (FFPE) tissues; the other 126 tumor specimens were obtained during surgery, then snap frozen in liquid nitrogen, and finally stored at −80°C until usage. The tumor specimens selected for DNA isolation were verified to consist of a minimum of 80% tumor cells in each case on hematoxylin and eosin‐stained tissue sections. All tumor specimens were histologically reviewed.

Data were collected on demographic characteristics, smoking status, alcohol intake, and family history of cancer. The definition of “ever smokers”, “never smokers”, “ever drinkers” and “never drinkers” could be found in *Appended Method*. Age at cancer diagnosis and additional clinical characteristics were extracted from medical records; TNM stages were defined according to American Joint Committee on Cancer Staging Manual, 7th edition 20.

Follow‐up was designed to carry out every 3 months for the first year; every 6 months for the next 2 years, and every 12 months from the fourth year after surgery. Information on event (death) and event time was obtained by trained nurses and medical students via telephone interview. For this study, the last follow‐up was performed on 31 July 2014.

### DNA extraction and quality control

Genomic DNA was isolated from frozen and FFPE samples using the TIANamp Genomic DNA Kit DP304 (Tiangen, Beijing, China) and TIANamp FFPE DNA Kit DP331 (Tiangen, Beijing,China), respectively.

PCR‐based approaches to analyze genes copy‐number changes in FFPE tissues have been successfully conducted 21, 22. As suggested 21, 22, only high‐ quality DNA template was accepted: (1) with an OD260/280 ratio range of 1.7–1.9, as measured by the NanoDrop 1000 instrument (NanoDrop Technologies, Wilmington, DE); (2) without DNA degradation (tested by Gel electrophoresis, Fig. S1). All DNA working solutions were diluted to 5 ng/*μ*L for final storage and usage.

### Primer design

By searching the DGV database (http://projects.tcag.ca/variation/?source=hg19), we found the landmark of *BCL2L1* CNV (chr20:30,252,261 .. 30,310,656), and then DNA sequence was downloaded from NCBI (http://www.ncbi.nlm.nih.gov/). Primer premier v5.0 software (Applied Biosystems, Foster City, CA, USA) was used to design primers for *BCL2L1*. The primers used for *MCL1* 19 were previously described.

Single‐copy genes *RPPH1* and *β‐globin* were used as reference genes. The primers for *RPPH1* were also designed by Primer premier, and the primers for *β‐globin* were extracted from published papers 23, 24, 25.

All primer sequences used in this study were available in Table S2. We subsequently conducted BLAST search (http://www.blast.ncbi.nlm.nih.gov) and UCSC In‐Silico PCR (http://genome.ucsc.edu) to evaluate and confirm the specificity of these primers.

### Real‐Time Quantitative Polymerase Chain Reaction

*BCL2L1* and *MCL1* CNVs were tested by real‐time quantitative polymerase chain reaction (RT‐qPCR) relative to two sing‐copy reference genes (*RPPH1* and *β‐globin*). To screen the CNV, combined use of two reference genes was suggested to produce a robust, reliable, and accurate quantification 26, 27, 28.

All PCRs were done on a 7900HT Sequence Detection System (Applied Biosystems) with Toyobo Thunderbird SYBR^@^qPCR Mix. The PCR reaction mixture (10 *μ*L) contained 5 *μ*L 2*×*SYBR mix, 3 *μ*mol/L each of the forward and reverse primers, and 3 *μ*L (5 ng/*μ*L) template DNA. All PCR reaction mixes were prepared in bulk with 10% extra volume, allowing excess for pipetting waste and were transferred to the 384‐well plate with multichannel pipette. The PCR conditions were 95°C for 5 min, followed by 40 cycles of 95°C for 15 sec, 58°C for 30 sec, and 72°C for 45 sec. Melting (dissociation) curve analysis was performed on every run to verify specificity and identify the PCR products. Test for *BCL2L1*,*MCL1*,*RPPH1*, and *β‐globin* were done in triplicate in the same 384‐well plate. The threshold cycle number (Ct) values were automatically determined by the ABI system.

Samples were successfully genotyped when Ct values for the triplicate fell within 0.3 units of each other; otherwise, the sample was retested. Triplicate wells of simplex (*BCL2L1, MCL1, RPPH1* or *β‐globin*) reactions containing a series of twofold dilutions of pooled germline DNA samples (50–1.0625 ng/reaction) from 50 randomly selected healthy individuals were used to determine the PCR efficiencies of each assay. Each plate also contained three negative controls (water) and calibrator DNA (this calibrator was verified to be without variations in these tested four genes by a whole genome copy‐number array in China). The r^2^ correlation for each standard curve was ≥0.98.

### Statistical analyses

OS was the primary outcome measure which was calculated from the date of operation to the date of death from any cause or the date of last follow‐up. Relative quantification of *BCL2L1*,* MCL1* was performed by the 2 ^‐∆∆Ct^ method 29 with little modifications as described by Lazar et al. 26, and the equation is shown in *Appended Method*. Consequently, *BCL2L1* or *MCL1* CNVs were dichotomized into two groups: “non‐amplification” and “amplification”. 2^‐∆∆Ct^ value >1 was interpreted as “amplification”. Multivariate Cox proportional hazards regression analysis was used to evaluate the effect of CNV status and clinicopathological variables on OS, illustrated as hazard ratio (HR) with their corresponding 95% confidence interval (95% confidence interval [CI]). The Kaplan–Meier method was used to compare OS among different CNV status. Statistical analyses were conducted with SAS software (version 9.1.3; SAS Institute, Cary, NC). Statistical significance of the improvement in area under curve (AUC) after adding an explanatory factor was calculated by the Delong's test 30. All statistical tests were two‐sided with a significance level of *a *=* *0.05.

### Bioinformatics analysis

CNV data and corresponding normalized gene expression data from tumor tissues for lung adenocarcinoma (LUAD) and Lung squamous cell carcinoma (LUSC) were obtained from The Cancer Genome Atlas (TCGA) data portal (http://cancergenome.nih.gov/, November 2014). This study meets the publication guidelines proved by TCGA (http://cancergenome.nih.gov/publications). The level 3 CNV segmentation data were retrieved and processed using TCGA‐assembler 31. If the CNV value was higher than 0.2, we defined it as “amplification”; otherwise, “non‐amplification”, as suggested by others scholars 32. Differences in gene expression levels between CNV status were assessed by a Wilcoxon signed‐rank test.

## Results

### Basic characteristics and genotyping data

The demographic and clinical variables of 516 patients with NSCLC are shown in Table**** 1. The patients were aged between 22 and 80 years at diagnosis with a mean of 58.01 years and standard deviation of 9.56 years. There were more men than women (389 vs. 127) and more ever smokers than non‐smokers (358 vs. 158); 250 of the patients had early‐stage lung cancer (stages Ia, Ib, IIa, and IIb). A total of 242 patients had lung squamous carcinoma, and 249 had lung adenocarcinoma. The median follow‐up time (MFT) for overall patients was 28 months, during when 234 (45.3%) patients died.

**Table 1:** Associations of patient demographic and tumor‐related characters with OS

Parameter	Patients	Death	MST	Univariate analysis^a^		Multivariate analysis^b^	
	No.	No.	(months)	HR (95% CI)	*P*	HR (95% CI)	*P*
Age (years)							
≤58	259	111	65.5	1.00		1.00	
>58	257	123	57.6	1.16 (0.9–1.50)	0.254	1.28 (0.97–1.69)	0.079
Sex							
Male	389	173	63.3	1.00		1.00	
Female	127	61	46.7	1.07 (0.80–1.43)	0.657	1.08 (0.67–1.76)	0.752
Smoking status							
Never	158	76	46.5	1.00		1.00	
Ever	358	158	63.9	0.91 (0.69–1.20)	0.494	1.05 (0.65–1.69)	0.842
Alcohol intake							
Never	311	139	61.6	1.00		1.00	
Ever	205	95	57.7	0.98 (0.75–1.27)	0.871	0.99 (0.72–1.36)	0.946
Family history of cancer							
No	440	202	59.1	1.00		1.00	
Yes	76	32	NA	0.89 (0.61–1.29)	0.538	0.86 (0.58–1.27)	0.451
Histological types							
Squamous Carcinoma	242	106	67.7	1.00		1.00	
Adenocarcinoma	249	124	46.5	1.22 (0.94–1.58)	0.137	1.37 (0.99–1.89)	0.055
Others	25	4	NA	0.29 (0.11–0.79)	0.015	0.26 (0.06–1.09)	0.065
TNM stage							
Ia	38	5	NA	1.00		1.00	
Ib	87	23	NA	2.34 (0.89–6.16)	0.085	2.63 (0.99–6.99)	0.053
IIa	62	24	NA	3.78 (1.44–9.92)	0.007	4.2 (1.59–11.13)	0.004
IIb	63	26	NA	4.70 (1.80–12.24)	0.002	5.24 (1.98–13.87)	0.001
IIIa	168	102	23.3	8.35 (3.40–20.53)	<.0001	8.68 (3.47–21.69)	<.0001
IIIb	28	21	16	12.64 (4.75–33.64)	<.0001	11.13 (4.07–30.48)	<.0001
IV	39	26	17	10.74 (4.12–28.05)	<.0001	10.21 (3.86–27.03)	<.0001
Missing	31						
Laterality							
Left	236	117	41.9	1.00		1.00	
Right	275	113	66.3	0.79 (0.61–1.03)	0.077	0.93 (0.71–1.22)	0.589
Others	5						
Surgical approach							
Lobectomy or sublobectomy	442	181	85.3	1.00		1.00	
Pneumonectomy	74	53	18.6	2.56 (1.88–3.49)	<.0001	1.82 (1.28–2.58)	0.001
Chemotherapy^c^							
No	209	87	NA	1.00		1.00	
Yes	307	147	48.9	1.07 (0.82–1.40)	0.613	0.87 (0.64–1.20)	0.398
Radiotherapy^c^							
No	372	156	84.8	1.00		1.00	
Yes	144	78	38.7	1.24 (0.95–1.63)	0.120	1.04 (0.75–1.44)	0.812
DNA source							
Fresh	126	33	NA	1.00		1.00	
FFPE	390	201	57.6	1.34 (0.92–1.95)	0.132	1.42 (0.97–2.09)	0.073

FFPE, formalin‐fixed paraffin‐embedded; MST, median survival time; OS, overall survival; HR, hazards ratio; 95% confidence interval, 95% CI; not available, NA.

a univariate analysis.

b Adjusted by other variables in Table 1.

c Chemotherapy or Radiotherapy after operation.

In multivariable analysis, TNM stage and surgical approach were found to be independent prognostic factors for NSCLC patients’ OS.

### CNV of BCL2L1, MCL1, and OS

**Figure 1:**
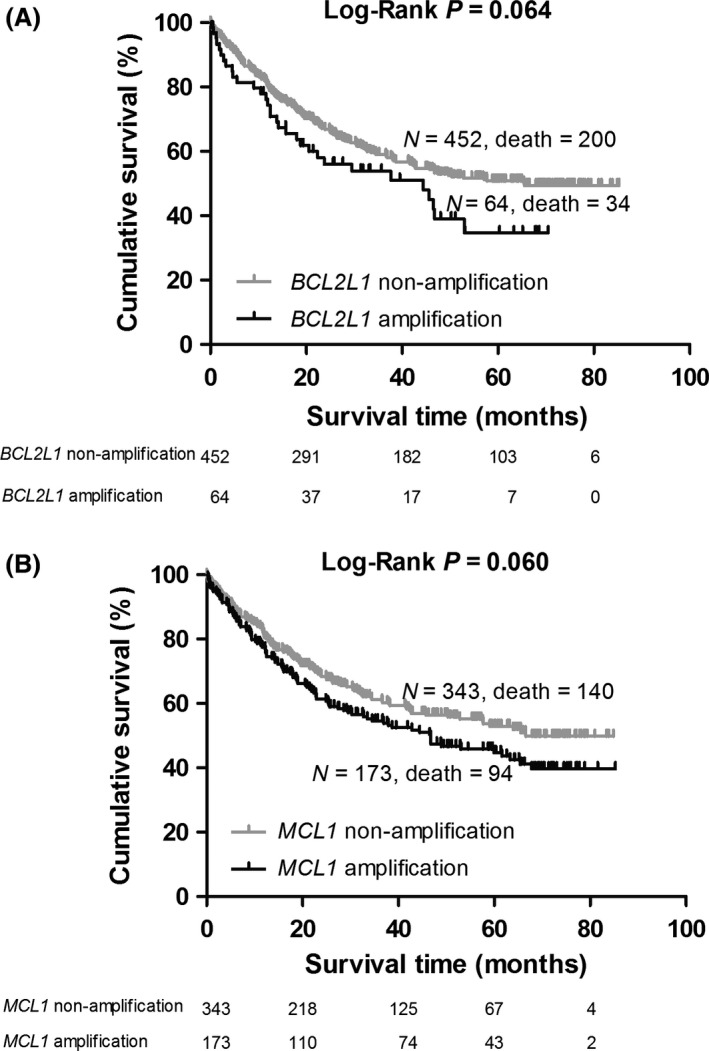
(A) Kaplan–Meier overall survival (OS) curve for patients without and with *BCL2L1* amplification; (B) Kaplan–Meier OS curve for patients without and with *MCL1* amplification.

In this study, 64 (12.4%), 173 (33.5%) of 516 NSCLC patients were identified to carry amplifications of *BCL2L1* and *MCL1*, respectively. Patients with amplifications of *BCL2L1* exhibited significantly increased hazards of death, compared with those without amplifications (adjusted HR = 1.62, 95% CI = 1.10–2.40, *P *=* *0.015, Table** 2 *)*. For *MCL1* CNVs, amplifications showed a strong association with shorter OS (amplification vs. non‐amplification, adjusted HR* *=* *1.39, 95% CI* *=* *1.05–1.84, *P *=* *0.020). For illustrative purpose, Kaplan–Meier curves of the associations with OS and CNV status are shown in Figure** 1.

**Table 2:** The Associations between CNVs *of BCL2L1, MCL1*, and NSCLC OS

CNVs	Number of		MST	Univariate analysis^a^		Multivariate analysis^b^	
	Patients	Death	(months)	HR (95% CI)	*P*	HR (95% CI)	*P*
*BCL2L1*							
Overall							
Non‐amplification	452	200	65.3	1.00		1.00	
Amplification	64	34	45.6	1.41 (0.98–2.03)	0.064	1.62 (1.10–2.40)	0.015
Squamous carcinoma							
Non‐amplification	214	89	NA				
Amplification	28	17	20.1	1.88 (1.12–3.17)	0.018	1.35 (0.76–2.39)	0.307
Adenocarcinoma							
Non‐amplification	221	108	45.3				
Amplification	28	16	37.6	1.36 (0.81–2.30)	0.251	1.62 (0.91–2.88)	0.102
*MCL1*							
Overall							
Non‐amplification	343	140	66.5	1.00		1.00	
Amplification	173	94	46.7	1.29 (0.99–1.67)	0.060	1.39 (1.05–1.84)	0.020
Squamous carcinoma							
Non‐amplification	170	69	NA				
Amplification	72	37	36.7	1.27 (0.85–1.89)	0.249	1.17 (0.77–1.80)	0.458
Adenocarcinoma							
Non‐amplification	158	68	57.2				
Amplification	91	56	38.0	1.31 (0.92–1.87)	0.139	1.40 (0.93–2.09)	0.105

CNVs, Copy‐number variation; MST, median survival time; OS, overall survival; HR, hazards ratio; 95% CI, 95% confidence interval.

a univariate analysis.

b Adjusted by age, gender, smoking status, alcohol intake, family history of cancer, histological types, TNM stages, laterality, surgical approach, chemotherapy, radiotherapy, and DNA source.

Different histological subtypes (LAUD and LUSC) have equal distribution of amplifications of *BCL2L1* (chi‐squared test, *P *=* *0.910) and *MCL1* (chi‐squared test, *P *=* *0.110). Subgroup analyses stratified by different histological subtypes showed that the prognostic effects of *BCL2L1* and *MCL1* failed to reach significance in the both groups of LAUD and LUSC (Table**** 2).

### Bioinformatics analysis

**Figure 2:**
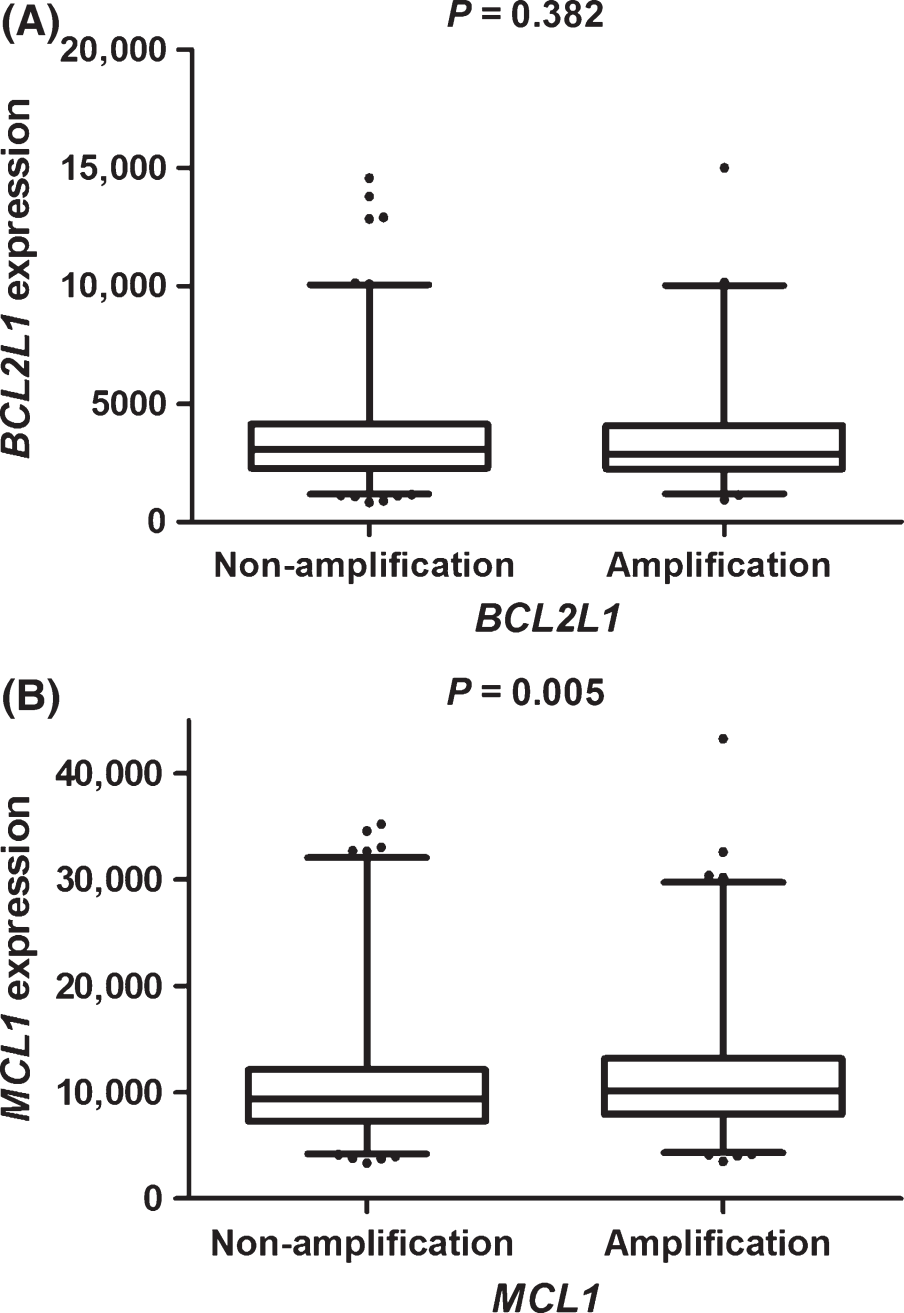
Analyses of *BCL2L1* (A) and *MCL1* (B) mRNA expression levels by corresponding CNV status in 986 NSCLC patients’ tumor tissues from the TCGA Project. CNV, copy‐number variation; TCGA, The Cancer Genome Atlas.

To explore the effect of CNVs in *MCL1* and *BCL2L1* on the corresponding genes expressions, we derived CNV data (in level 3) for tumor tissues from 491 LAUD and 487 LUSC patients in September, 2014., and normalized gene expression data (in level 3) from 1084 LAUD and 1033 LUSC tissues in TCGA database (performed on 17 November 2014). Overall, there were 986 NSCLC patients had data on both CNV and gene expression. As shown in Figure** 2, the amplification of *MCL1* was shown to be associated with higher expression levels of *MCL1* mRNA (*P *=* *0.005), compared with non‐amplification. Nevertheless, no significant correlation was found between *BCL2L1* CNV and mRNA expression levels (*P *=* *0.382).

### Receiver operating characteristic curve

**Figure 3:**
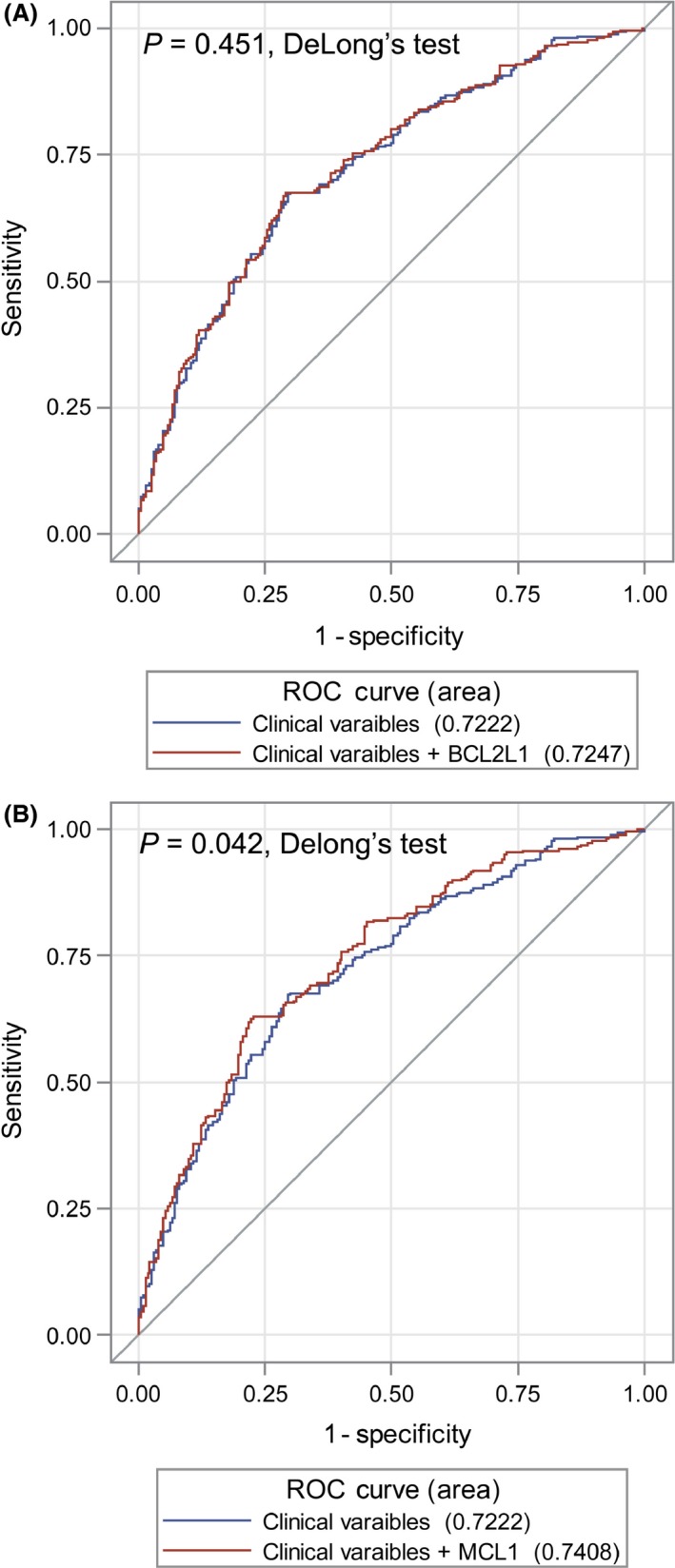
Receiver operating characteristic curves for prediction of OS rate based on clinical variables (age, sex, TNM stage, and surgical approach) and clinical variables plus *BCL2L1* copy‐number variation (CNV) (A) or *MCL1* CNV (B). OS, overall survival.

The capacity of CNVs for the classification of OS in NSCLC patients was evaluated by the multivariate logistic regression model and receiver operating characteristic curve. As shown in Figure** 3, with *MCL1* CNVs, the AUC for predicted model was significantly improved to 74.1%, which is higher than the 72.2% AUC for the model only included age, sex and the independent prognostic variables (TNM stage and surgical approach) (*P *=* *0.042, DeLong's test). But there is no similar finding for the *BCL2L1* CNV status (72.2% vs. 72.5%, *P *=* *0.451, DeLong's test).

## Discussion

In this study, we found that amplifications of *MCL1* and *BCL2L1* were likely to modulate OS of NSCLC patients in a southern Chinese population. However, only *MCL1* CNVs were found to be capable of influencing corresponding mRNA expression levels. The observed improvement of discrimination of NSCLC OS by *MCL1* CNV status supports the prognostic impact of associations and potential clinical applications.

CNV is proposed to influence gene expression, possibly by altering gene dosage, disrupting coding sequences, or perturbing long‐range gene regulation 33. Meanwhile, a study found that 53% of the expression probes associated with a comparative genomic hybridization (CGH) clone, were located outside the CNVs that encompass the specific clone 34. This suggested that gene expressions might be modulated by CNVs which were long‐range apart. Therefore, it is biologically possible that *BCL2L1* CNVs were not associated with corresponding mRNA expression levels.

*MCL1* CNV was found to influence mRNA expression levels of *MCL1*. MCL1 can bind and sequester the pro‐apoptotic proteins, Bax and Bak, and consequently suppress the release of cytochrome c from mitochondria into the cytoplasm 35. In cytoplasm, cytochrome c could induce the activation of caspases and then launch macromolecular degradation, which are the classic steps during mitochondrial pathway (intrinsic pathway) and apoptosis 36. *MCL1* plays a key role in cell immortalization, malignant transformation, and chemoresistance 16. Silencing the expression of *MCL1* with small interfering RNA (siRNA) potently killed a subgroup of NSCLC cell lines 17. Over expression of MCL1 protein was also found in a subset of human NSCLC cells. And high level of MCL1 may protect lung cancer cells from death induced by a variety of pro‐apoptotic stimuli 37. *MCL1* was suggested to be a critical molecule for chemoresistance in A549 cells associated with TGF‐*β*‐induced EMT 38. The expression of *MCL1* mRNA or protein has been associated with tumor progression and adverse patient outcome in multiple cancer types 39, 40, 41, 42, 43. BCL2 inhibitors could induce apoptosis; this has been explored as a therapeutic approach in NSCLC 44. A matter of interest is that, studies have suggested that MCL‐1 expression could be related to resistance to BCL2 inhibitors (such as ABT‐737) in lung cancer cell lines 45, 46. Therefore, *MCL1* CNV status might be considered for clinical decision of treatment using apoptotic inhibitor correlated with the Bcl‐2 proteins.

We are aware that this study has some limitations. First, relatively small sample size and short median follow‐up time were the major concern. However, by conducting power and sample size calculation 47, our study assessed *MCL1* CNVs in 516 NSCLC patients, achieving a power of 0.964 to detect the potentially clinical significant differences in OS. Second, patients in this study may have received a wide variety of therapies, often sequentially or simultaneously. Only information about whether patients received postoperational chemotherapy /radiation therapy was available. In this study, chemotherapy and radiation therapy did not independently affect NSCLC OS and there were no significant differences among results of subgroups that accepted variant chemotherapy and radiation therapy (data not shown). Third, the prognosis predicting model was only built in a Sothern Chinese population; the application and interpretation of our findings should be cautious and still needs further investigation. Fourth, lacking of information on resection margin status (R0 or R1) was another limitation. Finally, we acknowledge that this study will be more clinically critical if the association between the gene status and NSCLC‐specific death are explored, on which we continue to work.

Our findings suggested that the amplifications of *MCL1* might be associated with unfavorable NSCLC OS. However, the functional consequences of *MCL1* amplifications and external validations need to be extensively investigated in the future.

## Conflicts of Interest

The author(s) indicated no potential conflicts of interest.

## Supporting information


**Figure S1.** DNA degradation tested by Gel electrophoresis
**Table S1.** Characteristics of overall patients were recruited and patients were used in the current study
**Table S2.** Primer Sequence
