# Supplementary material for: Copy‐number variation of MCL1 predicts overall survival of non‐small‐cell lung cancer in a Southern Chinese population
Source: Cancer Med. 2016 Jun 5;5(9):2171–9. doi: 10.1002/cam4.774 (PMC4898974; doi:10.1002/cam4.774)
Supplement: Supplementary file 1 — Figure S1. DNA degradation tested by Gel electrophoresis Table S1. Characteristics of overall patients were recruited and patients were used in the current study Table S2. Primer Sequence [file CAM4-5-2171-s001.doc]

**Supplementary Material:**

**Copy number variation of *MCL1* predicts overall survival of non-small-cell lung cancer in a Sothern Chinese population**

Jieyun Yin^1,2^, Yangkai Li^3^, Hao Zhao^4^, Qin Qin^1^, Xiaorong Li^1^, Jiao Huang^1^, Yun Shi^1^, Shufang Gong^3^, Li Liu^1^, Xiangning Fu^3^, Shaofa Nie^1^, Sheng Wei^1*^

**Authors’ Affiliations:**

^1^ Department of Epidemiology and Biostatistics and MOE Key Lab of Environment and Health, School of Public Health, Tongji Medical College, Huazhong University of Science and Technology, Wuhan, China;

^2^ Department of Epidemiology and Biostatistics, School of Public Health, Medical College of Soochow University, 199 Ren-ai Road Industrial Park District, Suzhou, China;

^3^ Department of Thoracic Surgery, Tongji Hospital, Tongji Medical College, Huazhong University of Science and Technology, Wuhan, China;

^4^ Department of Social Science and Public Health, School of Basic Medical Science, Jiujiang University, No. 17, Lufeng Road, Jiujiang 332000, China.

***Corresponding Author: Sheng Wei,** Department of Epidemiology and Biostatistics and MOE Key Lab of Environment and Health, School of Public Health, Tongji Medical College, Huazhong University of Science and Technology, Wuhan 430030, China. Phone: 86 - 27 - 83692031; Fax: 86 - 27 - 83692031; E - mail: ws2008cn@gmail.com

**Supplementary method:**

1. Definition of “ever smokers”, “never smokers”, “ever drinkers” and “never drinkers”:

Those who smoked <1 cigarette per day and <1 year in their lifetime were defined as “never smokers”; otherwise, they were considered as “ever smokers”. “Ever drinkers” were defined as patients who had drunk at least one alcoholic beverage per week for at least 1 year during their lifetime, and patients who had never had such a pattern of drinking were considered “never drinkers”.

1. Gene ratio calculation :


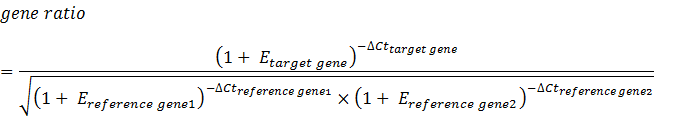


E _target gene_ is the efficiency of the PCR reaction for the target gene, E _reference gene_ is the efficiency of the PCR reaction for the reference gene,
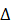
CT _target gene_ is the difference in threshold cycle value between the test sample and calibrator sample for the target gene,
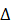
CT _reference gene_ is the difference in threshold cycle value between test sample and calibrator sample for reference gene.


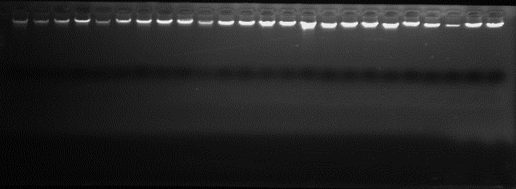


Supplementary Figure 1. DNA degradation tested by Gel electrophoresis.

| **Supplementary Table 1. Compare of the characters between the patients included this study and the overall patients in our sample bank.** | | | |
| --- | --- | --- | --- |
| **Variable** | **The patients included in this study**  **(n=516)** | **The sample bank**  **(n=1137)** | ***P**** |
| Age (years) |  |  |  |
| ≤58 | 259 | 543 | 0.385 |
| >58 | 257 | 594 |  |
| Sex |  |  |  |
| Male | 389 | 859 | 0.943 |
| Female | 127 | 278 |  |
| Smoking status |  |  |  |
| Never | 158 | 340 | 0.769 |
| Ever | 358 | 797 |  |
| Alcohol using |  |  |  |
| Never | 311 | 694 | 0.767 |
| Ever | 205 | 443 |  |
| Family history of cancer |  |  |  |
| No | 440 | 990 | 0.321 |
| Yes | 76 | 147 |  |
| Histological types |  |  |  |
| Squamous Carcinoma | 242 | 545 | 0.958 |
| Adenocarcinoma | 249 | 564 |  |
| TNM stage |  |  |  |
| Ia | 38 | 83 | 0.807 |
| Ib | 87 | 193 |  |
| IIa | 62 | 175 |  |
| IIb | 63 | 126 |  |
| IIIa | 168 | 378 |  |
| IIIb | 28 | 60 |  |
| IV | 39 | 91 |  |
| * Got from χ^2^ test. |  |  |  |

| **Supplementary Table 2.** Primer Sequence**.** | | |
| --- | --- | --- |
| **Gene** | **Forward primer(5’ - 3’)** | **Reverse primer(5’ - 3’)** |
| *BCL2L1* | GCC ATT TGT ATT CTT CAG AGC CA | CTA GGA GAA GGG CTG GCA AC |
| *MCL1* | CTT CCA AGG TAA GGG GGT TC | ACT GAC TCG TTT CGG TTT CC |
| *RPPH1* | CGT CCT GTC ACT CCA CTC CC | CCT GCC CAG TCT GAC CTC G |
| *β-globin* | CAC AAC TGT GTT CAC TAG C | CAA CTT CATC CAC GTT CAC C |
